# Bibliometric analysis of global research trends on ultrasound in inflammatory bowel disease: A quickly developing field

**DOI:** 10.1097/MD.0000000000042226

**Published:** 2025-06-06

**Authors:** Mengque Xu, Beibei Lin, Xingkang He, Qingyi Mao, Wenluo Zhang, Yu Zhang, Xiaoli Chen, Huiqin He, Xin Chen, Yu Zhang, Qian Cao

**Affiliations:** a Department of Gastroenterology, Sir Run Run Shaw Hospital, School of Medicine, Zhejiang University, Hangzhou, China; b Institute of Gastroenterology, Zhejiang University, Hangzhou, China.

**Keywords:** Bibliometric analysis, Bibliometrix, Inflammatory bowel disease, Ultrasound, VOSviewer

## Abstract

The aim of this study was to explore the scientific hotspots related to ultrasound in inflammatory bowel disease (IBD) through bibliometric approaches. The Web of Science Core database was used to identify articles about ultrasound and IBD that were published. We retrieved articles related to ultrasound and IBD from the Science Citation Index Expanded in the Web of Science Core Collection on March 7, 2024. The bibliometric analysis was carried out using Bibliometrix and the VOSviewer. The first article was published in 1979. The average annual growth rate of the publication count was approximately 10.24% during the study period. The USA (135 publications) and Italy (132 publications) were the mainstays in this field. Allocca Mariangela (24 publications) is a prolific author, yet Maaser Christian has the most citations. Regarding journals, the *Journal of Crohn’s Colitis* (68 publications) has the most publications on this topic. In terms of affiliations, the University of Milan (41 publications) has the highest number of publications. The co-occurrence analysis of keywords presented: diagnosis (intestine wall, activity index, etc), gastrointestinal ultrasound, management, pediatric patients. In recent years, “intestinal ultrasound,” “infliximab,” and “monitoring and healing” were the most active terms within these clusters. There has recently been a profusion of research on the application of ultrasound to IBD. Ultrasound examinations are beneficial tools in IBD diagnosis and assessment of treatment outcomes.

## 1. Introduction

Inflammatory bowel disease (IBD), which includes Crohn disease (CD) and ulcerative colitis (UC), is a chronic and destructive inflammatory disease of the gastrointestinal tract. IBD is one of the cornerstone topics in gastroenterology.^[1]^ For disease assessment during the diagnosis, treatment, and long-term follow-up of IBD, vast objective indicators are needed.^[2]^ These objective indicators include biomarkers, imaging data, endoscopy and pathology.^[3,4]^ A thorough assessment of the disease from diagnosis to follow-up is essential for selecting an appropriate treatment strategy for the patient and has a crucial impact on prognosis.^[5]^ A “treat-to-target” strategy is recommended for IBD management, which is based on the close monitoring of intestinal inflammation, including the activity of disease and response to treatment.^[6]^ Ileocolonoscopy and magnetic resonance enterography are currently the standard examinations for the assessment of both ileocolonic CD and UC.^[6]^ It has been suggested that magnetic resonance imaging (MRI) and/or intestinal ultrasound may be an better option for diagnostic evaluation of suspected IBD cases.^[2]^ Ultrasound, computed tomography (CT) and MRI all provide good judgement of intestinal stenosis due to IBD, and at least 2 modalities are more recommended for this judgement in the clinic.^[7]^

Ultrasound (US) is a commonly used clinical modality with the advantages of being cost-effective, noninvasive, free of ionizing radiation, well tolerated, and appealing to patients.^[8]^ The diagnosis of IBD with US relies heavily on the assessment of bowel wall thickness.^[9,10]^ US can also guide effective techniques for interventional procedures. For example, US-guided percutaneous or transrectal abscess drainage has a technical success rate of up to 96%.^[5]^ In addition, US can be used to determine the activity of the disease by detecting the density of the intestinal vessels.^[10]^ One meta-analysis showed that US had a sensitivity of 89.7% and a specificity of 95.6% in the diagnosis of IBD, with a mean specificity of 92.9% per bowel segment.^[10]^ Similar results were reported in another meta-analysis, in which the sensitivity and specificity of US in the diagnosis of CD ranged from 75% to 94% and 67% to 100%, respectively.^[11]^ The diagnostic accuracy was influenced by the thickness of the bowel wall. When the bowel wall thickness was >3 mm, sensitivity and specificity reached 88% and 93%, respectively; when the threshold was >4 mm, sensitivity and specificity reached 75% and 97%, respectively.^[11]^ In addition to conventional US, transabdominal intestinal ultrasound is helpful in the determination of IBD,^[12]^ and it has also been shown that image quality can be improved with oral intraluminal contrast agents to improve disease localization, sensitivity to intestinal complications and diagnostic accuracy.^[5]^ Contrast-enhanced ultrasound can improve the accuracy and confidence in the diagnosis of inflammatory activity.^[13]^ Endoscopic ultrasound (EUS) has a proven track record in assessing perianal lesions in patients with CD.^[14]^

Bibliometric analysis is a method of statistical analysis of scientific publications related to a particular topic. It is an important tool for obtaining scientific information quickly and assessing key research areas and new potential trends in future research.^[15]^ In recent years, some bibliometric analysis in the field of IBD has been reported and previous studies on related topics have been summarized and analyzed.^[16,17]^ Although the role of US in the diagnosis and treatment of IBD has significantly increased in recent years, few articles have provided a useful bibliometric analysis. In this study, we analyzed articles related to US and IBD that were retrieved from the Web of Science (WOS) core database and evaluated the available literature in terms of author, year, journal, country, institution, keywords, and references. The main aim of our study was to provide a broad understanding of the key themes and future directions of research related to US and IBD.

## 2. Materials and methods

### 2.1. Source and search strategy

We derived comprehensive literature documents from the Science Citation Index Expanded, Web of Science Core Collection (WOS Core database, Clarivate Analytics, United States). The search terms were “ultrasound,” “inflammatory bowel diseases,” and their synonyms, including “ultrasonography.” The specific search strategies used were as follows (TS=(“inflammatory bowel diseases” OR “crohn disease” OR “ulcerative colitis”)) AND TS=(“ultrasound” OR “ultrasonic” OR “ultrasonography” OR “ultrasonographic” OR “sonoelastography” OR “sonography” OR “sonographic” OR “ultrasonic sound” OR “supersound”).^[18]^ We searched all original article data during January 1, 1900, and December 31, 2023, and downloaded at March 7, 2024.

### 2.2. Inclusion and exclusion criteria

**Figure 1.**
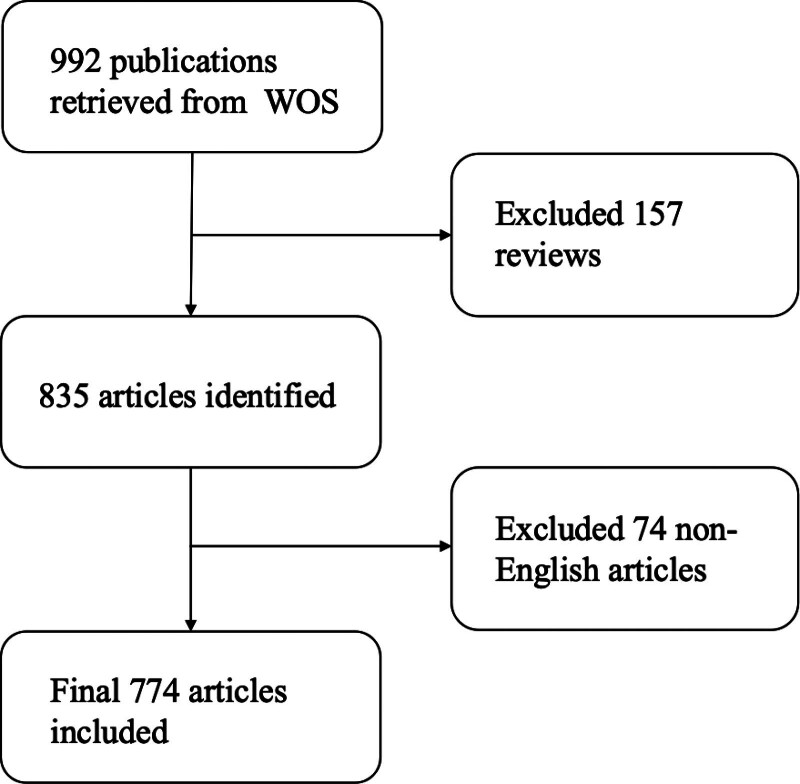
Flow chart of study inclusion on the topic of US and IBD. IBD = inflammatory bowel disease, US = ultrasound.

We included publications that described US in IBD. Both clinical and basic research were considered within the scope of this bibliometric analysis. Reviews and non-English articles were excluded. A detailed flowchart of the inclusion process is presented in Figure 1.

### 2.3. Statistical analysis

The original plain-text files containing the raw data of the cited sources were retrieved from the Science Citation Index Expanded, WOS Core database. We collected the title, author, institution, nation, year of publication, reference and keywords from among the raw data and saved them in TXT form. We imported the data into Rstudio “Bibliometrix” R package and analyzed annual publications, trends, distributions and frequencies for authors, journals, countries, and institutes. The data obtained from WOS were imported into VOSviewer version 1.6.20 (Leiden University) and were used to analyze co-authorship, co-cited references, and co-occurrence of keywords. Standard weight attributes were applied to define the “link attribute” and “total link strength attribute.” In the network, different nodes represent different elements such as authors, institutions, journals, countries/regions, keywords, and references. The size of the nodes represents the importance or frequency of different elements. The links between different nodes indicated the relationship between elements in terms of co-existence or co-citation. For the analysis using VOSviewer software, we applied its default threshold.

## 3. Results

Using the search strategies described above, a total of 992 papers were retrieved from the WOS. After the exclusion of 157 reviews, 835 research articles remained. To reduce bias due to language of publication, 74 articles that were not in English were excluded. The results of the data analysis were based entirely on information from the included 774 articles (Fig. 1).

### 3.1. Publication trends

**Figure 2.**
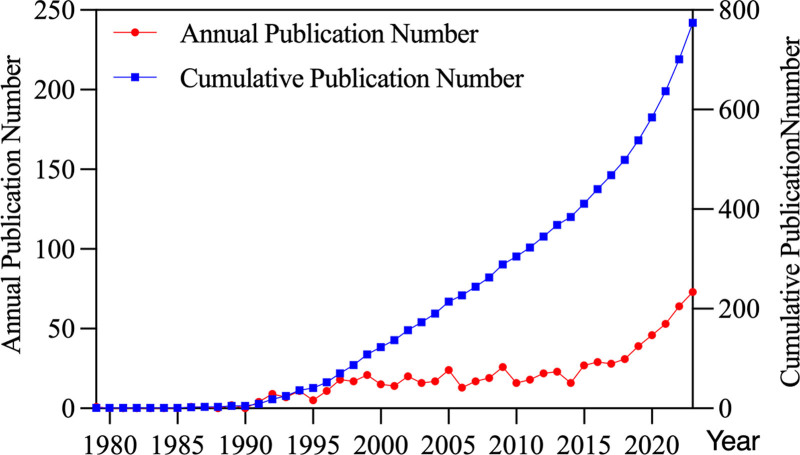
The patterns in the annual and cumulative numbers of publications in the period from 1979 to 2023.

The first article on the topic of US and IBD was published in 1979 by HOLT, S in the journal GUT, reporting that non-radioactive ultrasound contributes to the assessment of ileocaecal CD^[19]^. Before 1990, extremely few articles have been reported in this field. From this year onwards, a steady output of research in this field was produced through 2018. In the recent 5 years, this field has seen a boom in the number of publications year after year. The average annual growth rate of the number of publications from 1979 to 2023 is approximately 10.24%. In the last decade, the total number of published articles reached 406, accounting for 52.45% of the total number of articles in the last 5 decades (Fig. 2).

### 3.2. Analysis of authorship and co-authorship

**Figure 3.**
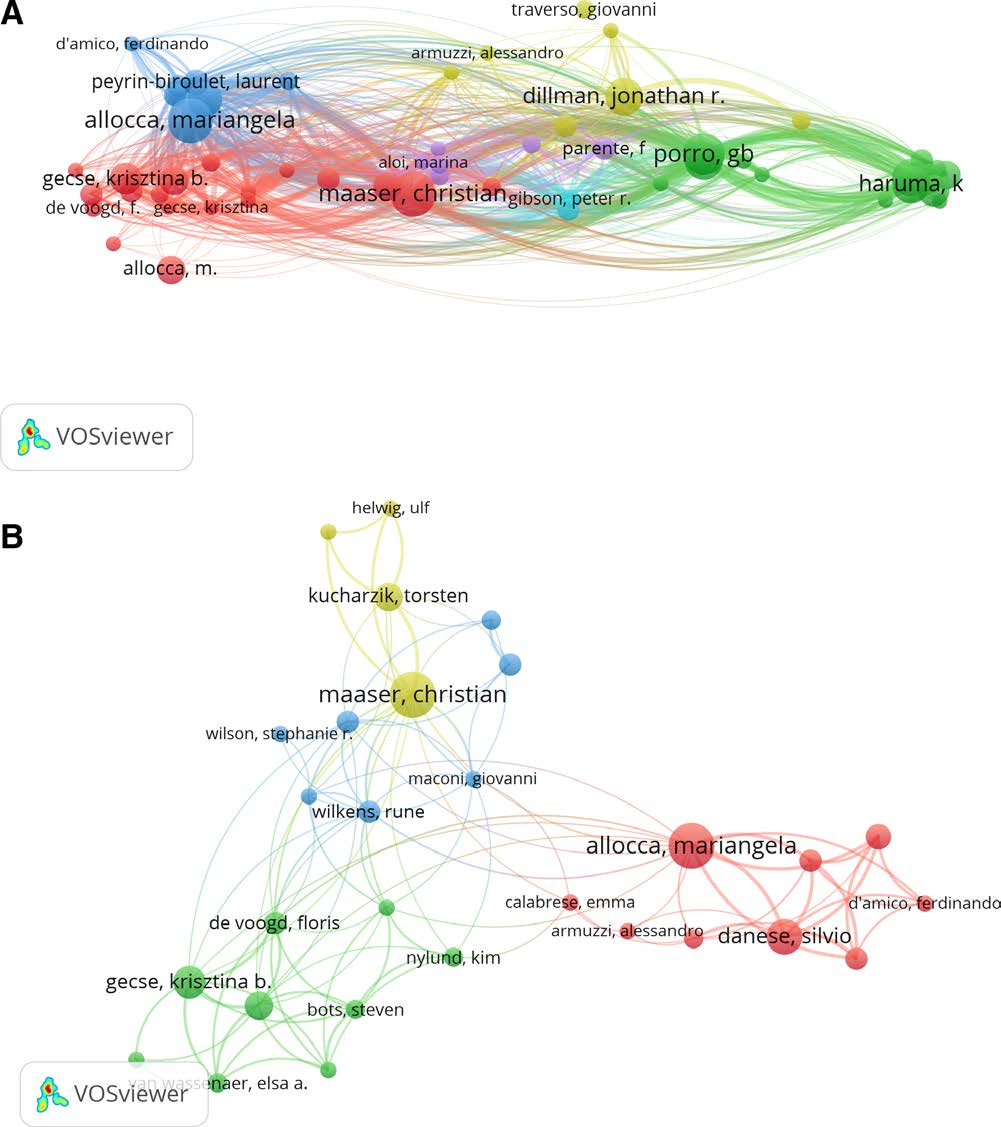
(A) Network of total numbers of author citations. (B) Network of co-authorship.

A total of 4051 authors were involved in the 774 articles. As shown in Table 1, the top 10 authors contributed a total of 171 articles (22.09%). Allocca Mariangela (24, 3.10%), was the author who contributed the highest number of articles. Other authors with significant contributions were Danese Silvio (21, 2.71%) and Maconi Giovanni (21, 2.71%). Among the most frequently cited authors, Maaser Christian was the most cited and influential author (Fig. 3A). Our analysis of authors’ co-authorship showed that the largest set of associated authors was included in the yellow cluster dominated by Maaser Christian, and the red cluster dominated by Allocca Mariangela (Fig. 3B).

**Table 1:** The top 10 productive authors in the field of “US and IBD”.

Author name	Publications	Author name	Publications
Allocca M	24	Fiorino G	15
Danese S	21	Furfaro F	15
Maconi G	21	Gecse KB	14
Maaser C	18	Peyrin-biroulet L	14
Porro GB	16	De Voogd F	13

IBD = inflammatory bowel disease, US = ultrasound.

### 3.3. Analysis of countries and affiliations

**Figure 4.**
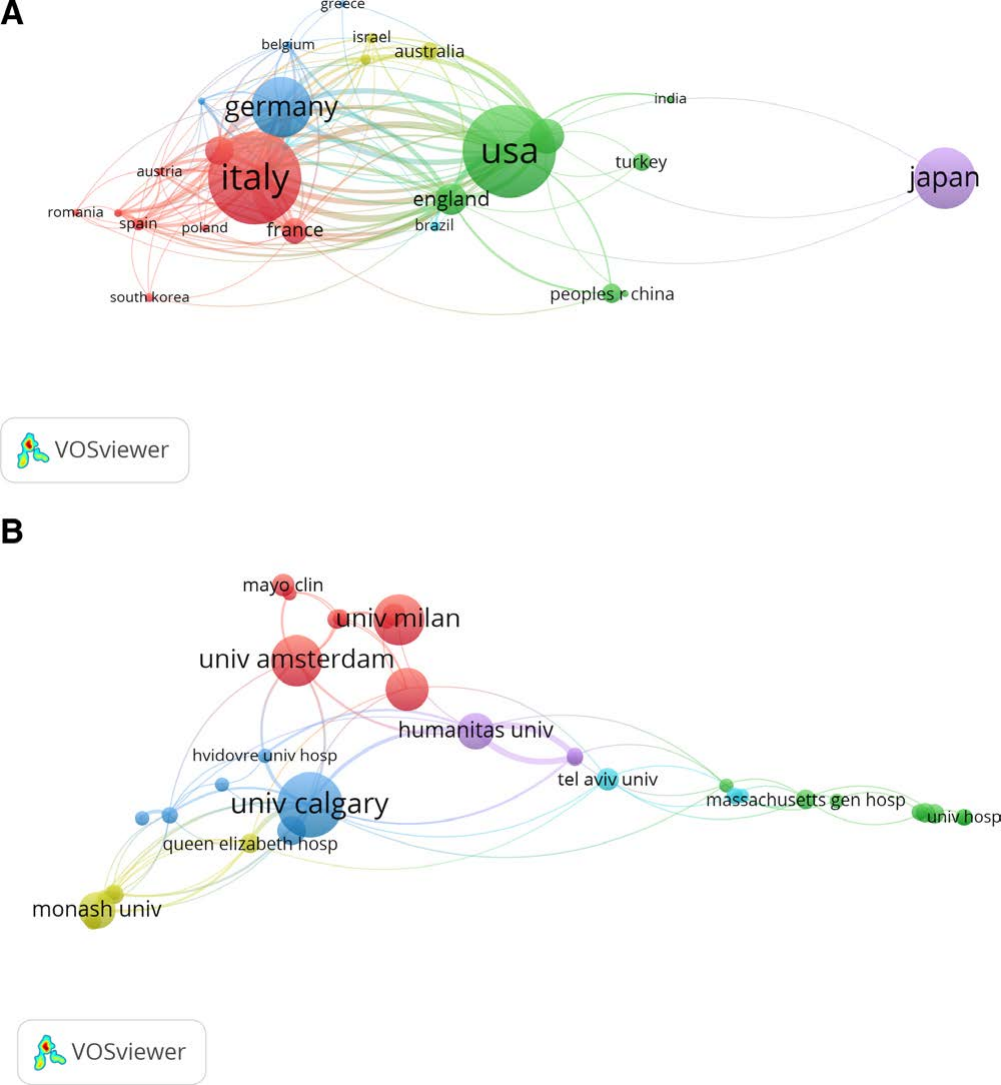
(A) Network map of co-authorship by country. (B) Network map of co-authorship by institution.

A total of 1111 affiliations from 53 countries/regions contributed to the publications on US and IBD. The top 10 countries/regions contributed 661 (85.40%) articles, with the top 5 rankings being the USA (135, 17.44%), Italy (132, 17.05%), Germany (85, 10.98%), Japan (85, 10.98%) and Canada (50, 6.46%) (Table 2). In recent years, Japan, Australia and Netherlands have had a boom in publications. A network map was created for the co-authorship analysis of countries and found that the United States, Italy, Germany, and Japan were the top 4 nodes (Fig. 4A). The top 10 affiliations contributed 209 (27.00%) of the number of articles, with the top 5 contributors being the University of Milan, Luigi Sacco Hospital, University of Amsterdam, the University of Calgary, and Harvard University (Table 2). The analysis of co-citations by institution suggests that the most 2 intensively cited are the red and green clusters, where the affiliations mentioned above, namely, the Luigi Sacco Hospital and the University of Calgary, are commonly co-cited (Fig. S1, Supplemental Digital Content, https://links.lww.com/MD/O700). The analysis of co-authorship by institution suggests that communication and collaboration of authors between different institutions to publish articles is common (Fig. 4B).

**Table 2:** The top 10 countries and institutions contributing to publications on the topic of US and IBD.

Country	Publications	Affiliations	Publications
USA	135	University of Milan	41
Italy	132	Luigi Sacco Hospital	28
Germany	85	University of Amsterdam	27
Japan	85	University of Calgary	23
Canada	50	Harvard University	19
England	45	University of London	17
Netherlands	40	Humanitas University	14
France	36	University College London	14
Peoples r China	27	Academic Medical Center Amsterdam	13
Turkey	26	Monash university	13

IBD = inflammatory bowel disease, US = ultrasound.

### 3.4. Analysis of journals

As shown in Table 3, the journals that have published the most studies on the topic of US and IBD include the *Journal of Crohns Colitis* (68, 8.79%), *Gastroenterology* (42, 5.23%), *Inflammatory Bowel Diseases* (38, 4.91%), *Scandinavian Journal of Gastroenterology* (25, 3.23%), *World Journal of Gastroenterology* (22, 2.84%), among others. These data reflected the interest of journal editors and reviewers in this area of research, providing researchers with more options for submission.

**Table 3:** The top 10 most published journals in the field of “US and IBD”.

Journals	Publications
Journal of Crohns Colitis	68
Gastroenterology	42
Inflammatory Bowel Diseases	38
Scandinavian Journal of Gastroenterology	25
World Journal of Gastroenterology	22
Alimentary Pharmacology Therapeutics	20
Pediatric Radiology	19
Gastrointestinal Endoscopy	18
Journal of Ultrasound in Medicine	18
Radiology	18

IBD = inflammatory bowel disease, US = ultrasound.

### 3.5. Co-cited keyword co-occurrence cluster analysis

**Figure 5.**
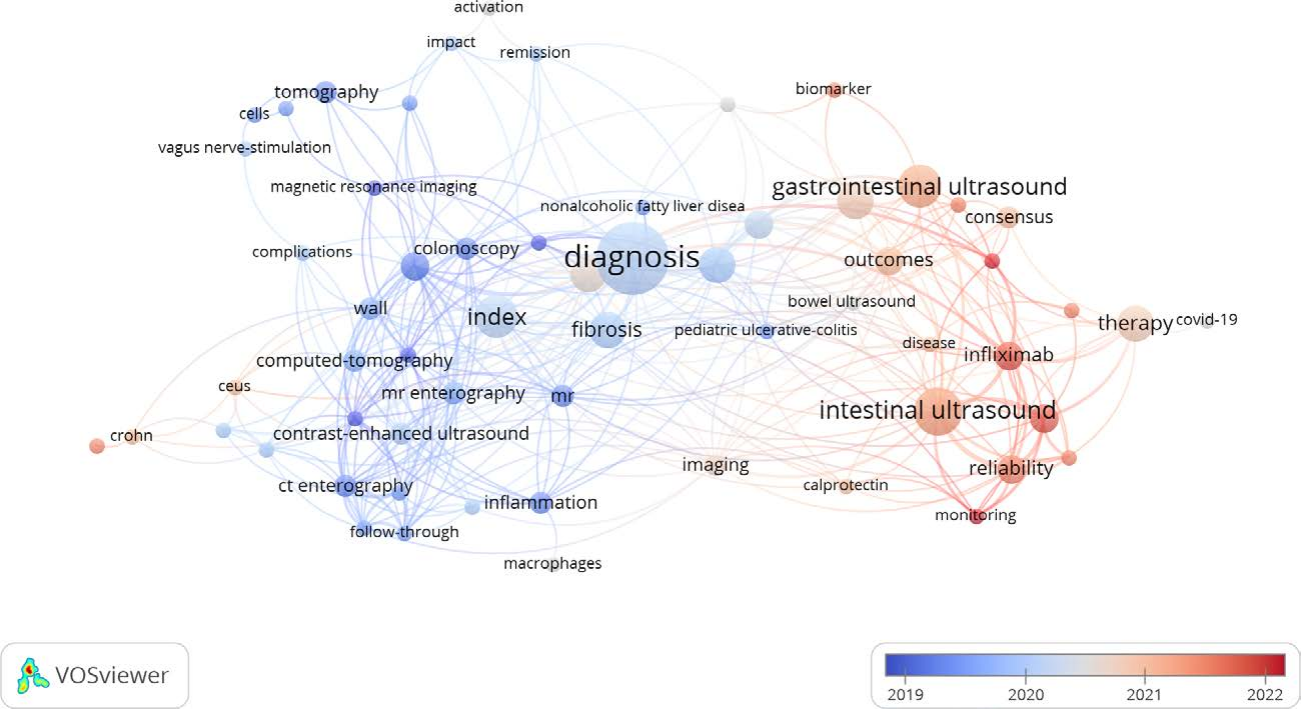
The network map of keywords in recent 5 years.

The keywords were extracted from the publications and mapped for network visualization (Fig. S2, Supplemental Digital Content, https://links.lww.com/MD/O700). The cluster analysis shows 7 clusters, such as purple cluster related to diagnosis (intestine wall, activity index, etc), blue and light blue clusters related to different kinds of US and the management of IBD, the green cluster related to chlidren’s diagnosis and management, and yellow cluster related to other diagnostic tests (CT, endoscopy, etc). The larger size of the nodes indicates more mentions as keywords and more weight and influence, such as diagnosis, management, children and intestinal ultrasound. We analyzed the keyword hotspots in the past 5 years and found that “intestinal/gastrointestinal ultrasound,” “infliximab,” and “monitoring and healing” were the largest among the red dots in the graph, which indicates that these are the most active research directions in recent years (Fig. 5).

### 3.6. Co-cited reference analysis

**Figure 6.**
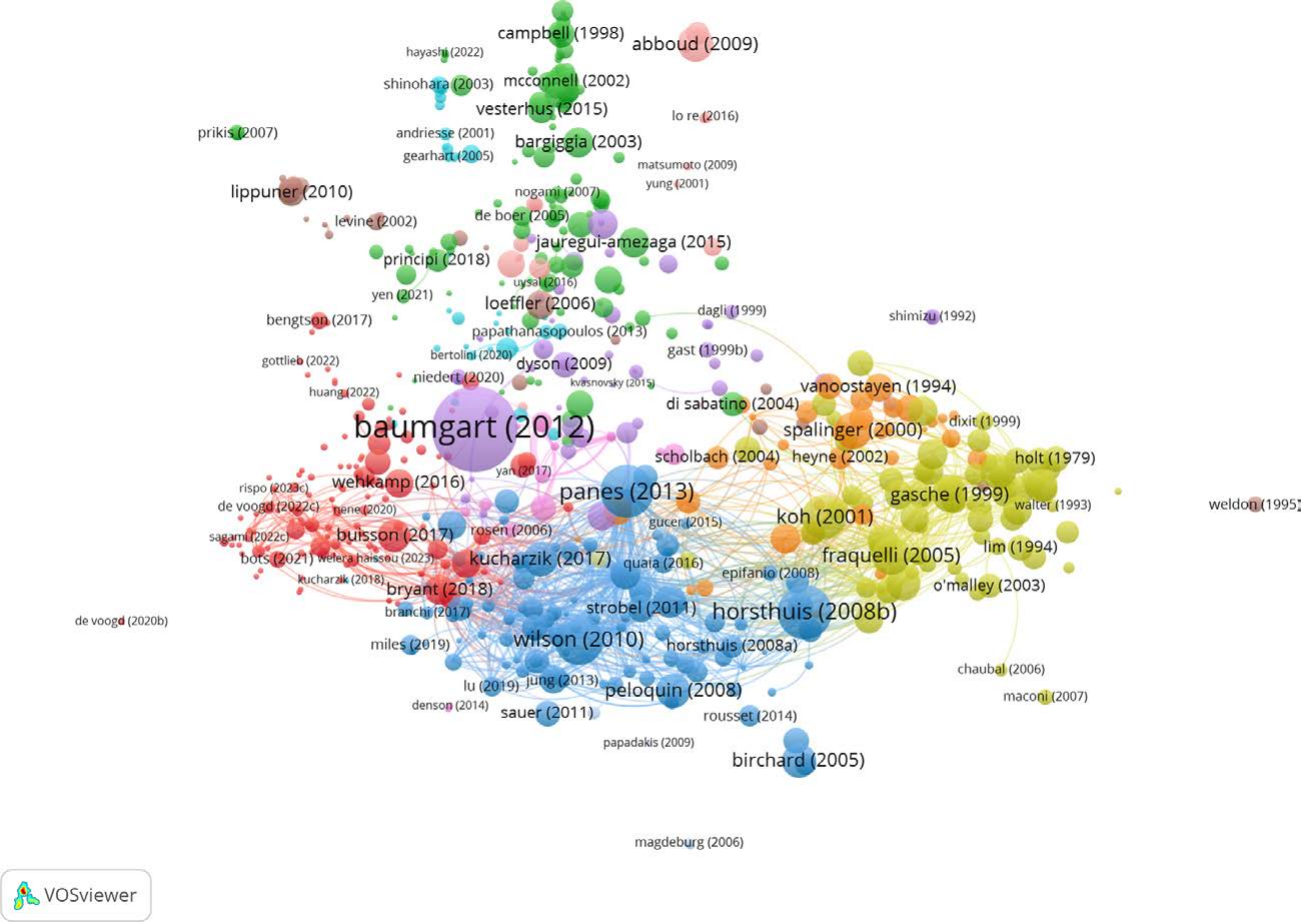
The network map of publications.

Table 4 presents the top 10 cited articles in this field. As shown in Table 4, the article published by Baumgart DC et al has been cited more than 1400 times, and the weight and influence of this article are also visible in Figure 6. Most of the other articles in Table 4 have been cited 150 to 450 times. The co-cited references were clustered, and a network diagram was created to visualize these publications. Five main clusters are depicted in Figure 6, with the highest 3 of references in the red, green, and blue cluster.

**Table 4:** The top 10 most frequently cited references on the topic of US and IBD.

Titles	First author	Journal	Year	Citations
Crohn disease	Baumgart DC	*Lancet*.	2012	1431
Inflammatory bowel disease diagnosed with US, MR, scintigraphy, and CT: meta-analysis of prospective studies	Horsthuis K	*Radiology*	2008	451
Imaging techniques for assessment of inflammatory bowel disease: joint ECCO and ESGAR evidence-based consensus guidelines	Panes J	*J Crohns Colitis*	2013	439
Microbubble-enhanced US in body imaging: what role	Wilson SR	*Radiology*	2010	348
MR imaging evaluation of the activity of Crohn disease	Koh DM	*AJR Am J Roentgenol*	2001	270
Role of US in detection of Crohn disease: meta-analysis	Fraquelli M	*Radiology*	2005	204
Doppler US in patients with crohn disease: vessel density in the diseased bowel reflects disease activity	Spalinger J	*Radiology*	2000	184
Transabdominal bowel sonography for the detection of intestinal complications in Crohn disease	Gasche C	*Gut*.	1999	166
Hepatic portal venous gas: physiopathology, etiology, prognosis and treatment	Abboud B	*World J Gastroenterol*	2009	159
Abdominal ultrasound in the assessment of extent and activity of Crohn disease: clinical significance and implication of bowel wall thickening	Maconi G	*Am J Gastroenterol*	1996	159

IBD = inflammatory bowel disease, US = ultrasound.

## 4. Discussion

In this study, we performed a bibliometric analysis of the search results in the WOS core database to identify relevant trends and hotspots in the study of US and IBD. Our results revealed something that can be adopted by researchers. It is well known that the annual number of academic publications is an important indicator of trends in the field. Before 1990, the number of publications in this field was poor. Since then, the number of publications in this field has grown vigorously. This phenomenon indicates that the field has attracted the attention of many scholars in recent years due to the fast pace of research. In earlier studies, researchers were more interested in exploring the sensitivity and specificity of ultrasound in the identification of IBD.^[20,21]^ Coincidentally, many articles focused on the use of EUS in IBD, and in recent 15 years, there has been a proliferation of research reports in this area. EUS can help diagnose IBD.^[22]^ Endoscopic US elastography can improve the diagnostic accuracy of IBD by assessing the stiffness of the intestinal tissue.^[23]^ Ultrasound is of great benefit to pediatric patients because of its freedom from radiation. “Children”/”Paediatric” is also a buzzword in this field (Fig. 5A and Fig. S2, Supplemental Digital Content, https://links.lww.com/MD/O700). Ultrasound is widely used in the clinic to assess IBD disease status in pediatric patients. More retrospective or prospective studies and systematic reviews comparing the accuracy and specificity of ultrasound for IBD would enable many patients to reduce the number of radiological or invasive endoscopic investigations.^[12]^ However, ultrasonography is dependent on the skill and subjective judgement of the operator and needs to be regulated by clearer industry guidelines. Due to the advantages of low-radiation and noninvasive characteristics of ultrasound, it has gained prominence in the diagnosis and follow-up of IBD in pediatric patients in recent years.^[24]^ Similarly, the importance of ultrasound in adult IBD has been emphasized, which has relevant systematic analyses and guidelines published in recent 5 years.^[12,25–27]^

In terms of citations to publications, Maaser Christian is the most frequently cited and influential author, and Allocca Mariangela is the author with the most publications. High-impact publications are focused on the use of US to diagnose IBD, assess disease remission and manage disease follow-up. The analysis of the national origin of the publications reveals a remarkable contribution of the United States in this field, as well as a significant contribution from Italy. The distribution of affiliations coincides with the above data on national sources. Two Italian institutions, namely, the University of Milan and Luigi Sacco Hospital, are the leading institutions in the study of US and IBD. According to the results of the keyword analysis, US, especially gastrointestinal ultrasound, has greater research potential in the assessment of treatment outcomes and disease management.^[25–28]^ The above information is intended to provide the readers with an understanding of the respective fields and to give more emphasis to the publications of these authors, organizations and countries, which may suggest important information in the field of “IBD and ultrasound.”

This study based on bibliometric tools provides more quantitative and comprehensive information on research priorities, trends, and collaborations than traditional literature reviews, providing better insight into areas of future development in the study of US and IBD. However, our report has several drawbacks. First, our literature was sourced from the WOS core database only, excluding non-English language articles, which may have introduced bias into the inclusion of publications. However, to ensure the high quality of the raw data obtained, we chose to include only the WOS core database. We chose to exclude non-English articles in order to ensure the consistency and accuracy of the data analyzed in terms of authors, institutions, keywords, and other information. Second, because the article citation data are limited by time, the citation counts of some recent publications may be severely underestimated. The data of some excellent new publications may be obscured under this method. This relies on continued attention and bibliometric analysis of the field.

## 5. Conclusion

In conclusion, this study offers a bibliometric analysis to examine the dynamics in publications on US and IBD throughout the last 5 decades. It presented trends in the number of publications, important authors and hot keywords, which may provide a valuable understanding and brief summary of the current status and trends of research in this field. Further high-quality clinical trials and state-of-the-art reviews are warranted to boost the citation count and conclusively establish the role of US applications in IBD. US is a valuable examination tool in the diagnosis and efficacy evaluation of IBD.

## Author contributions

**Conceptualization:** Mengque Xu, Beibei Lin.

**Data curation:** Beibei Lin, Xingkang He, Qingyi Mao.

**Formal analysis:** Mengque Xu, Beibei Lin, Xingkang He.

**Funding acquisition:** Mengque Xu, Yu Zhang.

**Investigation:** Beibei Lin, Wenluo Zhang, Yu Zhang, Xiaoli Chen, Huiqin He, Xin Chen.

**Methodology:** Beibei Lin, Xingkang He.

**Project administration:** Mengque Xu, Yu Zhang, Qian Cao.

**Resources:** Mengque Xu, Yu Zhang.

**Software:** Beibei Lin, Xingkang He.

**Supervision:** Mengque Xu, Qian Cao.

**Validation:** Mengque Xu, Qian Cao.

**Visualization:** Beibei Lin, Xingkang He.

**Writing – original draft:** Mengque Xu, Beibei Lin.

**Writing – review & editing:** Mengque Xu, Qian Cao.

## Supplementary Material


