# Supplementary material for: Bibliometric analysis of global research trends on ultrasound in inflammatory bowel disease: A quickly developing field
Source: Medicine (Baltimore). 2025 Jun 6;104(23):e42226. doi: 10.1097/MD.0000000000042226 (PMC12150968; doi:10.1097/MD.0000000000042226)

Supplementary Figure 1. Network map of co-cited institutions.


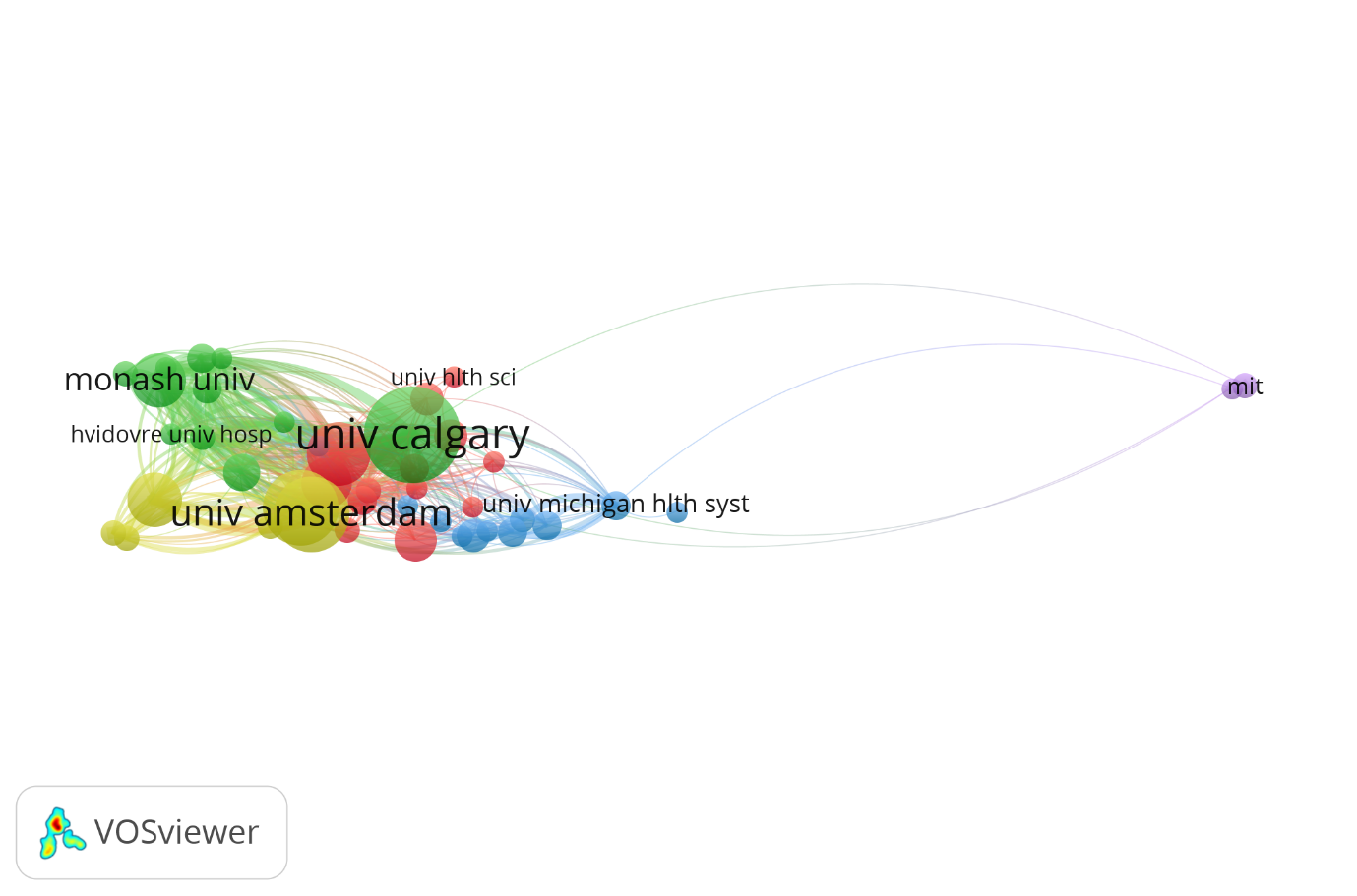


Supplementary Figure 2. The network map of keywords.


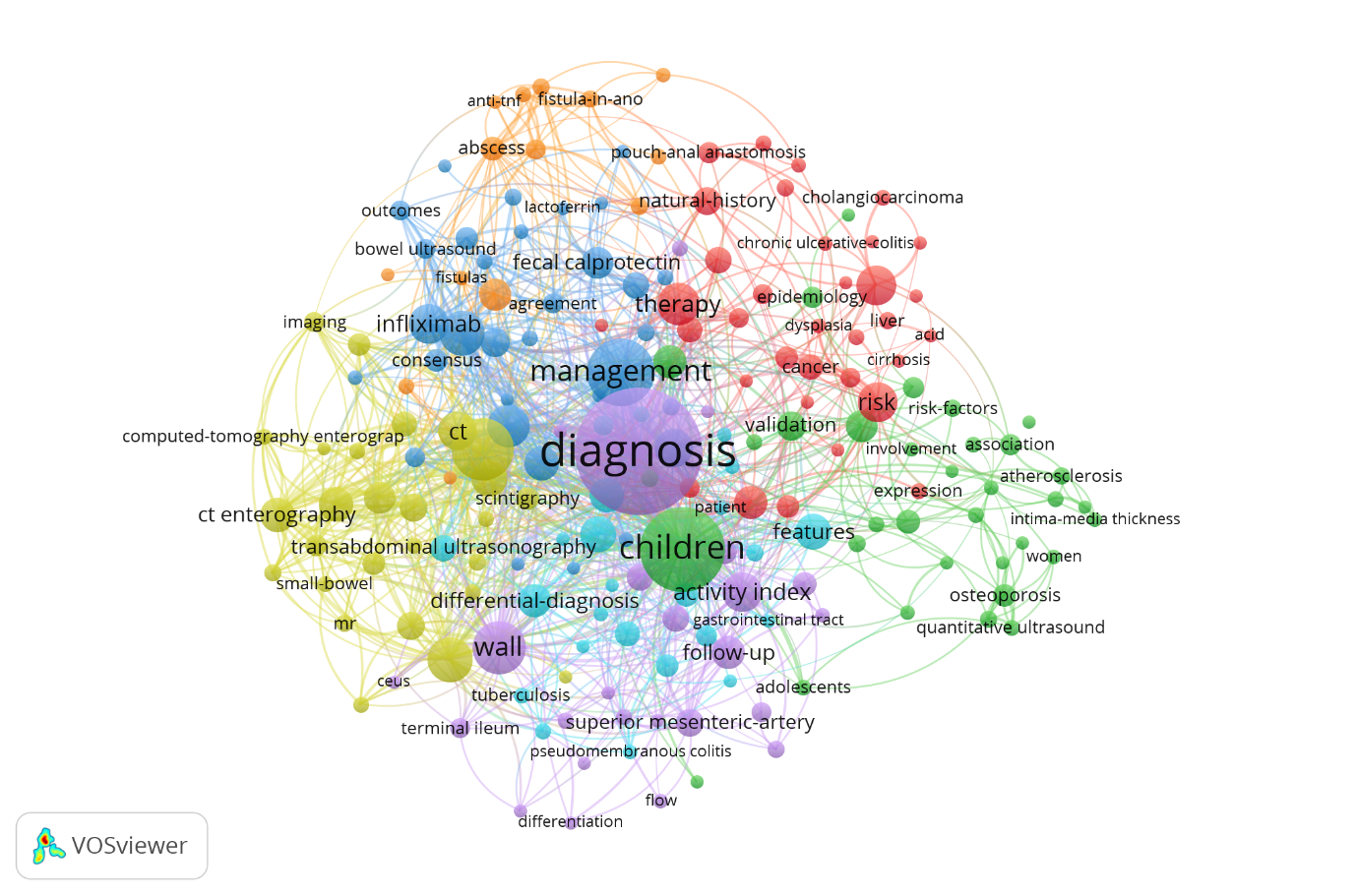

Supplement: Supplementary file 1 [file medi-104-e42226-s001.docx]
